# Adsorption of Azo Dye Methyl Orange from Aqueous Solutions Using Alkali-Activated Polypyrrole-Based Graphene Oxide

**DOI:** 10.3390/molecules24203685

**Published:** 2019-10-13

**Authors:** Abdulaziz Ali Alghamdi, Abdel-Basit Al-Odayni, Waseem Sharaf Saeed, Mohammed S. Almutairi, Fahad A. Alharthi, Taieb Aouak, Abdullah Al-Kahtani

**Affiliations:** Chemistry Department, College of Science, King Saud University, P.O. Box 2455, Riyadh 11451, Saudi Arabia; aalghamdia@ksu.edu.sa (A.A.A.); 436105200@student.ksu.edu.sa (M.S.A.); fharthi@ksu.edu.sa (F.A.A.); taouak@ksu.edu.sa (T.A.); akahtani@ksu.edu.sa (A.A.-K.)

**Keywords:** polypyrrole-based activated carbon, alkali activation, adsorption, methyl orange, water pollution, dye removal

## Abstract

The adsorption of methyl orange (MO) from aqueous solutions onto a KOH-activated polypyrrole-based adsorbent (PACK) was investigated using batch and fixed-bed column techniques. The structural, thermal, and morphological properties of the PACK, analyzed by various methods, support its applicability as an adsorbent. An adsorption kinetic study revealed a preferably pseudo-second-order (*R*^2^ = 0.9996) and rate-limiting step controlled by both film and intra-particle diffusions. The thermodynamic adsorption tests resulted in negative ΔG°, ΔH°, and ΔS° values, which decreased as the temperature and concentration increased, indicating the spontaneous and exothermic adsorption over 25–45 °C. The adsorption isotherms fit the experimental data in the order of Langmuir ≈ Freundlich > Temkin, with evidence of adsorption operating well via the monolayer physical adsorption process, and maximum monolayer adsorption ranging from 520.8 to 497.5 mg/g. The breakthrough curve of the fixed-bed column experiment was modeled using the Thomas, Yoon–Nelson, and Hill models, resulting in an equilibrium capacity of 57.21 mg/g. A 73% MO recovery was achieved, indicating the possibility of column regeneration. Compared to other adsorbents reported, PACK had comparable or even superior capacity toward MO. For cost-effectiveness, similar nitrogen-containing polymeric wastes could be exploited to obtain such excellent materials for various applications.

## 1. Introduction

Dye pollution is a global issue owing to the hazardous nature of dyes. It is considerably visible in the environment, as dyes are commonly highly water-soluble and not readily degradable under natural conditions, resulting in a relatively long-time residence in the environment. Therefore, the removal of dyes from various toxic effluents has become of special concern to environmental specialists. Azo dyes such as methyl red and methyl orange (MO) are well known to be human carcinogens [1] that pervade aqua systems from various sources, including textile, pharmaceutical, and printing industries, as well as medical and chemical labs. Although most dyes are not highly toxic, they should be recognized as visual pollutants that can reduce light penetration into water, consequently decreasing the efficiency of photosynthesis and affecting the growth of aquatic organisms [2]. Moreover, dye pollution presents aesthetic changes that are harmful in the natural environment. Methyl orange (MO) is an anionic, water-soluble (5 g/L, H_2_O, 20 °C) azo dye, which can function as a weak acid having an approximate pH of 6.5 when dissolved in water. Besides its use for industrial coloring, it also serves as a pH indicator in various laboratories, with a red to yellow color-change range of 3.1–4.4. MO is a sulfonated azo dye, and the functional groups that are responsible for their bright color in water make them difficult to remediate through conventional methods. Dyes bearing aromatic amines in their chemical structures are carcinogenic due to the production of benzidine compounds via biotransformation [3].

Various physical–chemical methods have been employed for the removal of dyes from wastewater, including filtration, adsorption, precipitation, coagulation, and photo-degradation [3]. Among these, the adsorption technique is the most competitive method, with a number of advantages, such as low cost, high efficiency, and simple operation [4]. In this technique, different adsorbents from various sources are explored to find the best material, subsequently, a convenient adsorption system is built. These adsorbents are comprised of microorganisms, activated carbon, zeolite, biomasses, polymers, and polymer-based adsorbents [5]. Among the available adsorbents, activated carbon is the most effective owing largely to its high surface-area, internal porous structure, and varying surface functional group, as well as its simplicity, high efficiency, and cost-effectiveness [6,7]. The production of carbon materials can be accomplished through a programmed heating process of the carbon precursor. Basically, there are two methods for activation: physical and chemical. The former requires a relatively high temperature, and the chemical activation requires a low temperature, which can be accomplished in the presence of acid, base, or salt.

Due to the limitations in the adsorption efficiency associated with each type of adsorbents, no single adsorbent has been found to be effective for the removal of different types of pollutants. This necessitates a continual effort to overcome such a challenge in this field. In this context, the prepared polypyrrole-based activated carbon (PACK), in this work, was reported to exhibit promising properties, including a high surface area and appropriate pore sizes. Moreover, its gas storage application, sensing properties, and the removal of metal ions from their aqueous solutions have been investigated, and based on the results, PACK is an effective adsorbent [4,8,9].

The present work was designed to test the feasibility and adsorption performance of PACK as a promising adsorbent for the removal of MO, a representative dye pollutant, from synthetic aqueous solutions in batch and fixed-bed column systems. The adsorption kinetic behavior, thermodynamic, and isothermal adsorption properties as well as the column efficiency were evaluated using different models, and the best adsorption-describing parameters were determined and reported.

## 2. Results and Discussion

### 2.1. Characterization of the Adsorbent

**Figure 1:**
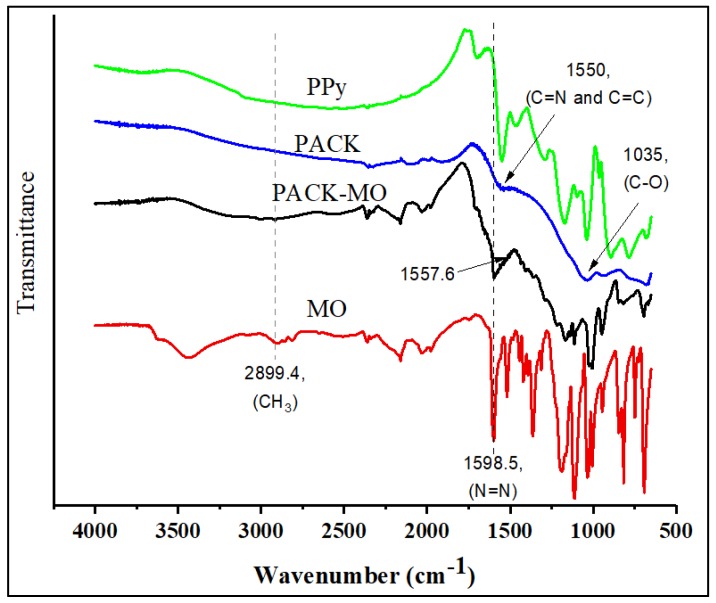
Fourier transform infrared (FTIR) spectra of Polypyrrole (PPy), KOH-activated polypyrrole-based adsorbent (PACK), PACK–MO, and methyl orange (MO).

**Figure 2:**
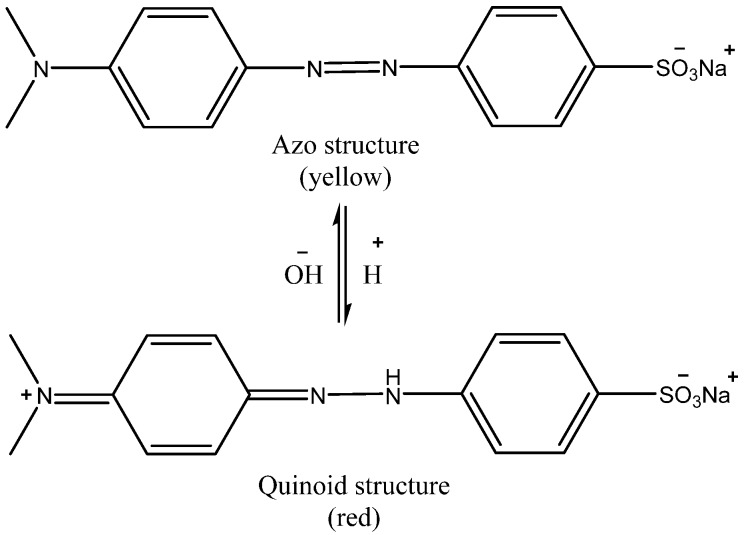
Chemical structure of MO dye: the acidic and basic forms.

The PACK adsorbent was prepared following the method from a previous work [4]. Its features, including the surface texture, thermal stability, and crystallinity, were also reported [4,8,9]. The SEM micrographs of Polypyrrole (PPy) (shown in Supplementary Data, Figure S1), revealed a cauliflower-like typical surface morphology, while the PACK image indicates a rough and peel-like structure having a surface with large, irregularly shaped pores, with an average internal diameter of 2.2–4.2 µm. To gain insight into the elemental composition for the material under investigation, elemental analyses using both carbon, hydrogen and nitrogen (CHN) and energy-dispersive X-ray (EDX) methods were performed and compared with the reference [9] (Table for elemental compositions and selected EDX images of PPy and PACK are given in the Supplementary Data (Figures S1–S3)). However, the PACK C/N ratios were found to be as low as 11.3 (CHN method result) compared to the literature (55.2) [9], indicating high nitrogen content. The Fourier transform infrared (FTIR) spectra of PACK before and after the adsorption process are shown in Figure 1 along with the PPy spectrum for comparison. The structural differences between the material under investigation were noticeable. Therefore, the spectrum of PPy was in a good agreement with those reported in the literature [10], in which the fundamental peaks of PPy-ring were observed at 1548.9 and 1470.7 cm^−1^, the =C–H in-plane vibration at 1285.9 and 1038.7 cm^−1^, and the C–N stretching at 1170.3 cm^−1^. The apparent absence of an O–H peak in the PACK spectrum indicated a reduced structure. The existing of a peak around 1550 cm^−1^ (reported at 1556 cm^−1^ [11]) is due to the superposition of the vibrations of both C=N and C=C, and is an evidence of the embedding of nitrogen-containing groups [11]. However, a peak at about 1035 cm^−1^ attributed to C–OH (or epoxy group) is still present, revealing a partial reduction, furthermore, no evidence for the presence of C=O exists. The peaks at 930.4 and 679.9 cm^−1^ may be attributed to the ring deformation and physisorbed carbon dioxide, respectively [12]. The MO FTIR spectrum is similar to the reported one [13]. After adsorption of MO by PACK, the peaks assigned to C=N (about 1550 cm^−1^) and C–O (1035 cm^−1^) in the PACK spectrum shifted slightly toward higher values (1557.6 and 1195.8 cm^−1^), suggesting new types of interactions of each of the nitrogen and oxygen functional groups of PACK in the presence of MO. Indeed, additional peaks were observed in the PACK–MO spectrum due to the attachment of MO molecules. The chemical structure of MO in acidic and basic media is depicted in Figure 2.

**Figure 3:**
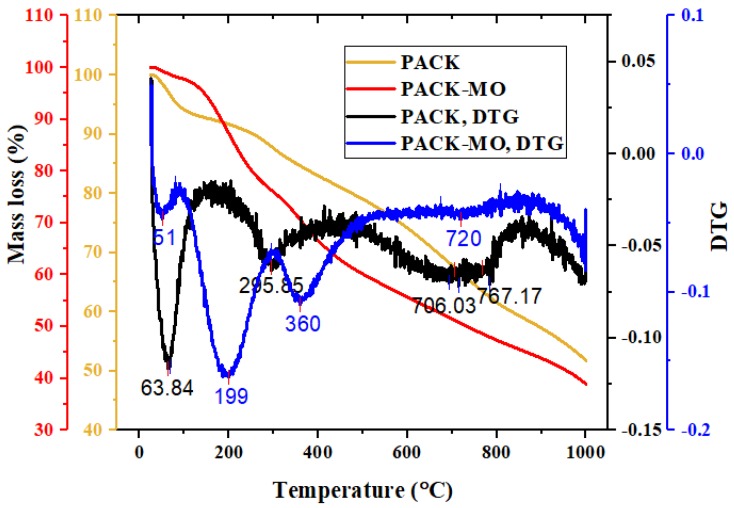
Thermogravimetric analysis (TGA) thermograms of the adsorbent before (PACK) and after adsorption (PACK–MO), and the corresponding derivative curves (DTGs).

The thermogravimetric analysis (TGA) shown in Figure 3 indicate the multistep degradation of PACK before and after the adsorption of MO (Table 1). The observed total mass loss of 48.2% of PACK before the adsorption may indicate the incomplete carbonaceous property, however, volatiles were lost first below 100 °C with a mass loss of 5.6%. After that, a significant step around the derivative-TGA (DTG) value of 295.8 was observed with a total mass loss of 8.6%. Thereafter, the mass loss continued to degrade gradually up to 1000 °C, with a slight drop after 650 °C, which may be due to the reduction in the presence of nitrogen atmosphere. Compared to PACK thermogram, the PACK–MO thermogram also exhibited almost three steps of degradation with distinct differences due to the presence of MO attached to its surface. Evidently, the mass loss due to the MO molecules is between approximately 100 and 400 °C. The relatively high mass loss (19.1%) between 100 and 265 °C may indicate more adsorption of MO into the external surface compared to the internal surface, which is possibly lost between 265 and 445 °C (15.6%).

**Figure 4:**
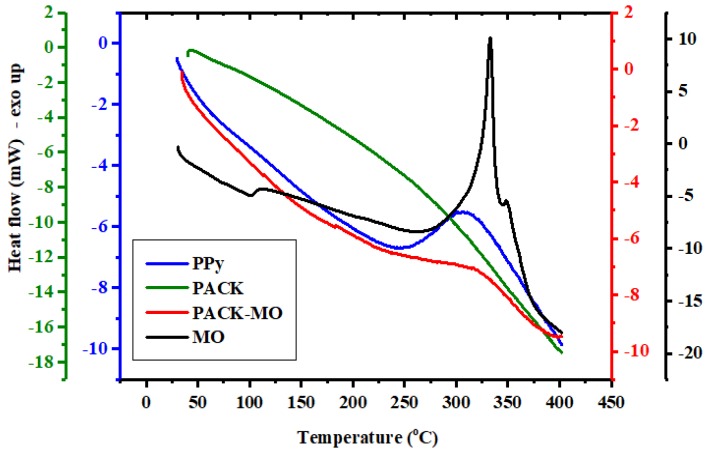
Differential scanning calorimetry (DSC) thermograms of PPy, PACK, MO, and PACK–MO.

Figure 4 shows the differential scanning calorimetry (DSC) profiles of PPy, PACK, MO, and PACK–MO. The PPy exothermic peak at 305 °C (onset 245 °C) may indicate that a chemical reaction has taken place, involving bond cleavage, depolymerization, crosslinking, and decomposition. The DSC curve of PACK shows no clear heat transitions in this temperature range. However, the endothermic trend became sharper at the approximate onset of 250 °C, which is consistent with the TGA second-step of decomposition. Two exothermic peaks of MO centered at 330 and 349 °C were observed indicating its instability above 270 °C, i.e., decomposition before melting [14]. After adsorption, the attached MO molecules onto PACK resulting in an exothermicity at 320 °C (onset approximately at 240 °C), assuming early degradation of MO stimulated by carbon.

### 2.2. Adsorption Kinetic

**Figure 5:**
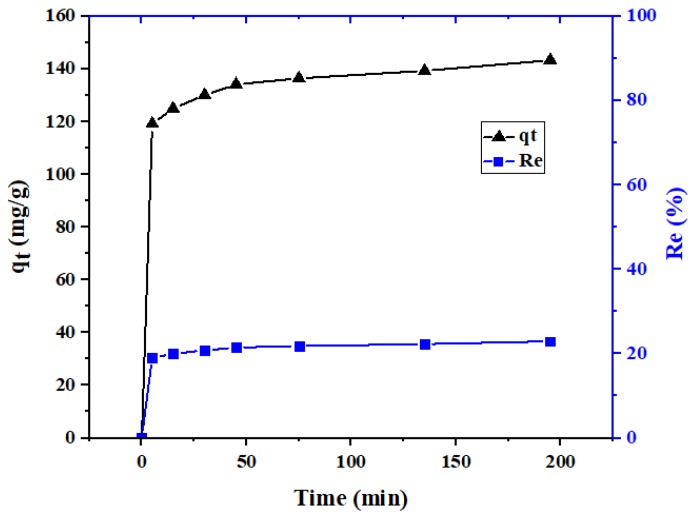
Effect of contact time on the adsorption of MO onto PACK.

**Figure 6:**
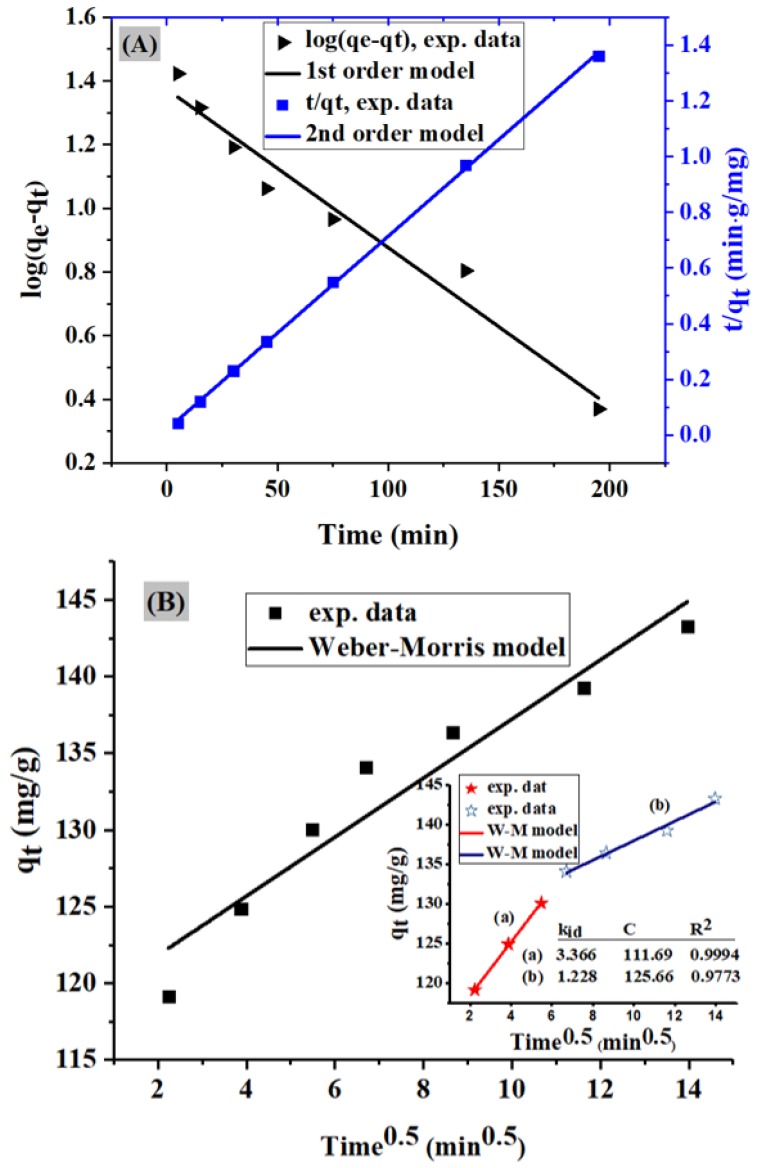
(**A**) Pseudo-first-order (left axis) and pseudo-second-order (right axis). (**B**) The intra-particle diffusion plot for the MO adsorption; the insert is a two-stage intra-particle diffusion. Adsorbate C_0_ = 50 mg/L, volume = 500 mL, adsorbent = 0.04 g, temperature = 23 ± 2 °C, and agitation speed = 150 rpm.

Figure 5 shows the time-dependent adsorption behavior of MO by PACK. In this figure, the rapid adsorption in the initial period (5 min) reveals that the adsorption mainly occurs on the surface of the adsorbent, assuming the chemisorption mechanism. After that, the adsorption increased slowly, showing practically negligible increase after 195 min (reference samples were measured after 24 h) assuming a physisorption mechanism, which may be controlled by the diffusion process [15]. Commonly, a high number of adsorbent vacant sites are available for the MO molecules to be adsorbed in the first stage of the adsorption, after which repulsive forces, as well as the diffusion mechanism, may be dominant, thereby reducing the adsorption rate. Thus, the mechanism involved in the mass transfer of the MO dye from liquid phase to the PACK surface was evaluated using the pseudo-first-order (PFO) (Equation (11)) and pseudo-second-order (PSO) (Equation (12)) kinetic models (Figure 6A). By ignoring the adsorbate movement from the bulk liquid to the adsorbent near the liquid film, three stages of the adsorption process can be defined: (1) the outer (or boundary layer) diffusion, the external adsorbate mass transfer across the liquid film to the adsorbent exterior surface; (2) the inner (or intra-particle) diffusion, which is the transport of adsorbate particles from the adsorbent exterior surface to its internal pores; and (3) the interaction process [16], which is the step that is very fast and thus cannot be treated as rate-limiting. To determine the actual rate-controlling step involved in the adsorption process, the intra-particle diffusion model described by the Weber–Morris (W–M) equation (Equation (14)) was also applied (Figure 6B) Table 2. The relatively high correlation coefficient value of the PSO (*R*^2^ = 0.9996) compared to that of the PFO (*R*^2^ = 0.9639) as well as the proximity of the adsorption capacity (q_e_) (143.89 mg/g) to the experimental one (143.38 mg/g) of the PSO model indicate that the adsorption system is a PSO reaction. The linear plot of the W–M model passing through the origin suggests the intra-particle diffusion, while its deviation from the origin suggests the participation of other mechanisms in the rate-controlling step, including the film diffusion mechanism. The high value of C (117.98 mg/g) indicates a large boundary effect, thus, this external film resistance cannot be ignored. Due to the mass-transfer effects, the intra-particle plot is curved at a small time-limit (from 30 to 45 min), below and above which the intra-particle diffusion model operates efficiently (*R*^2^ = 0.9994 and 0.9773, respectively). Since the slope (k_id_) of the first stage is greater than that in the final stage, the diffusion model mainly controlled the uptake of MO onto PACK, as shown in the plot of the two stages (insert in Figure 6B) [17].

### 2.3. Adsorption Isotherms

Table 3 summarizes the isotherm parameters expressed by the Langmuir, Freundlich, and Temkin linearized equations. Based on the data obtained, the applicability of the three isotherms for the present data was in the following order: Langmuir > Freundlich > Temkin. The values of the 1/*n* and the heterogeneity factor (R_L_) indicate favorable adsorption. As the value of 1/*n* approaches zero, more heterogeneous conditions are expected. The maximum adsorption capacity (q_m_, mg/g) obtained from the Langmuir equation was relatively decreased from 520.8 to 497.5 mg/g as the temperature increased from 298 to 318 K, indicating a less favorable adsorption at relatively high temperatures. Moreover, this case is confirmed by the ∆G° magnitudes, which approach less negative values at relatively high temperatures. In addition, the K_F_ values predict a decrease in the adsorption capacity as the temperature increases, thereby supporting this argument. The Temkin equilibrium binding constant (A), corresponding to the maximum binding energy, decreased as the temperature increased from 298 to 318 K, indicating that the adsorption process is exothermic [18]. Hereinafter, the adsorption, preferably spontaneous and exothermic, is operated well, based on the monolayer physisorption process.

### 2.4. Adsorption Thermodynamics

The thermodynamic parameters can be obtained from a curve established between the apparent equilibrium constant K_0_ (Equation (9)) versus 1/T according to the Van′t Hoff equation (Equation (8)) (Table 4). The negative values of ∆G° and ∆H° show that the adsorption of the MO dye is spontaneous and exothermic. However, the temperature-dependent ∆G° change was trivial, showing a slight decrease as the temperature increased from 298 to 318 K, although a considerable decrease with concentration increase was observed. The negative ∆S° value suggests a decrease in the randomness at the solid–liquid interface during the adsorption of the MO dye on PACK [19]. Moreover, a decreasing trend of the ∆H° and ∆S° values was observed. Generally, the value of ∆G° for physical adsorption (from −20 to 0 kJ/mol) is smaller than that for chemisorption (from −80 to −400 kJ/mol) [20]. Similarly, a ∆H° value of less than 40 kJ/mol indicates the physical process [21].

Based on the above results, it could be concluded that the adsorption reaction mechanism is PSO, monolayer, physical, spontaneous, exothermic, and less favorable at high temperatures.

### 2.5. Column Behavior

**Figure 7:**
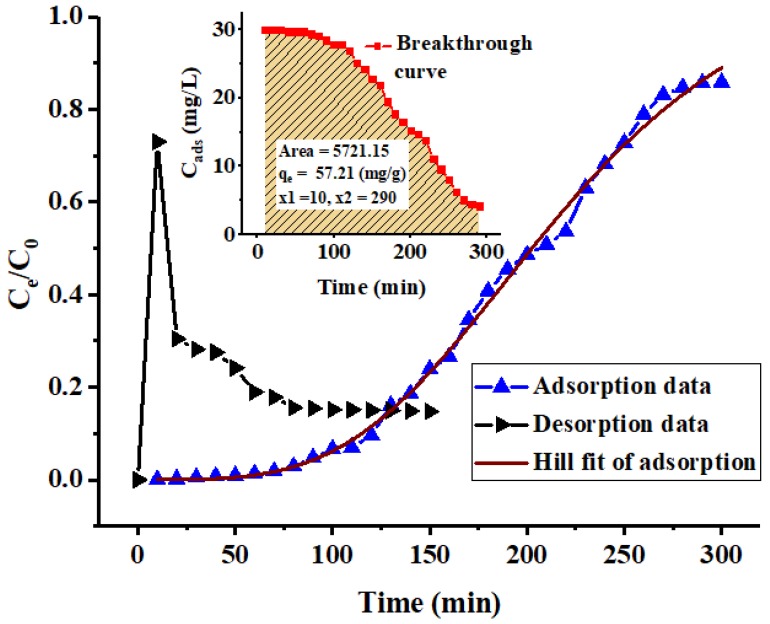
Experimental breakthrough curve and Hill model fit of the fixed-bed column adsorption process, and the desorption curve. The inset shows the area of the integrated curve of adsorption.

The applicability of the PACK column adsorption of MO was evaluated through the breakthrough curve analysis generated by plotting the quantity of MO adsorbed (C_ads_, mg/L) or the normalized values of the effluent concentration (C_e_/C_0_), against the time (t, min), resulting in a noticeable S-shaped curve (Figure 7), from which the performance of the established column system can be evaluated. Using the given data, the adsorption capacity (q_e_) (mg/g) of the column can be calculated as in Equation (1) [22]:

(1) $$q_{e}=\frac{\mathrm{QA}}{1000\;m}$$

where A is the breakthrough curve area, Q is the volumetric flow rate (mL/min), and m (g) is the mass of the adsorbent in the column. Accordingly, the experimental capacity at equilibrium (q_e_. Exp.) was found to be 57.21 (mg/g), and the breakthrough point (C_e_/C_0_ = 0.05) and exhaustion times were evidently at 90 and 290 min, respectively, under the applied operation conditions (MO C_0_ = 30 mg/L, PACK bed amount = 0.1 g, flow rate = 1 mL/min, and operation temperature = 23 ± 2 °C). Moreover, the breakthrough curve was fitted to the three-parameter logistic model (also known as the Hill model) (3-PL) (Figure 7), and the data obtained are listed in Table 5. Despite the goodness fitting (*R*^2^ = 9968) of this model to the experimental data, it reflects no much information, therefore, it cannot be used for designing the fixed-bed column. The predicted inlet concentration (b value, 1.17) is, however, close to unity. The value of c, the time taken to attain a 50% inlet concentration, was predicted as 219.8, which is not considerably far from that determined value by the Yoon–Nelson model (209.2). Commonly, the value of the parameter (c) depends on many factors, including the bed height, initial concentration, and flow rate. The steepness determiner (the Hill slop *n*) was found to be 3.67, which may reflect the degree to which the adsorbate diffusion delayed due, supposedly, to the intra-particle diffusion or ion-exchange before the column exhaustion. However, the *n* value is in the range reported for some data analysis [23].

Despite the limitation of this experimental part, the intended fundamental information concerning the feasibility of the designed column has been acquired. Nevertheless, adequate data have been obtained through modeling the breakthrough curve using the Thomas, Yoon–Nelson (Y–N), and Hill models. The results indicate that both models are suitable for describing the column adsorption behavior, indicated by the reasonable value and equality of the regression coefficient (*R*^2^) of 0.9676. The theoretical Thomas value of the bed capacity, q_e_, was higher than the experimental value (69.7 and 57.2 mg/g, respectively), indicating an adequate adsorption condition including the initial MO concentration and flow rate for optimal uptake. Both the Y–N and 3-PL models predicted relatively close values for the time of 50% uptake: 209.1 and 219.8 min, corresponding to the τ and c parameters, respectively.

Figure 7 also shows the regeneration curve of the PACK adsorbent. In the adsorption step, around 87% MO removal was achieved, of which around 73% was recovered in the regeneration step during 80 min. However, about 66% was desorbed at the first 20 min. The relatively low regeneration percentage may be due to the insufficient NaOH, which allowed some MO molecules to remain in the binding sites. However, surface destruction and possibly other effects may be responsible for low reusability, this may necessitate further experiments.

The maximum monolayer adsorption capacities, q_m_ (mg/g), of some other absorbents for the MO dye uptake obtained from literature are listed in Table 6 for comparison. As can be seen, the value of q_m_ of the investigated PACK for MO adsorption is higher than that of the other potential adsorbents listed in the table. However, the differences of the MO adsorption capacities are due to the diversity in the adsorbent characteristics concerning its chemical structure and the surface texture, such as the surface area, porosity, and functional groups. To select the most suitable adsorbent, many factors have to be considered, such as the availability, reusability, and cost-effectiveness. The adsorbent produced from naturally available materials including agricultural, biosource, industrial waste, or readily naturally available ones may be less expensive, but less efficient. By some modification, their capacity to remove dye pollutants may be increased. Synthetic materials including activated carbon have shown enhanced adsorption depending on the source as well as the type of treatment and method of synthesis. Nitrogen-containing adsorbents may have resulted in an elevated efficiency owing to the presence of nitrogen, which offers beneficial sites for the electrostatic interaction with charged adsorbates. Regeneration, however, is the major drawback associated with the potential adsorbents. In most cases, it is not done or not disclosed in publications. Conversely, the regeneration trials of some adsorbents have resulted in a low-performance percentage as can be seen in the listed examples. Comparing the column capacity at equilibrium (q_e_, mg/g) may provide indication about the column efficiency. For this reason, data from some columns used for the removal of MO from its aqueous solutions are tabulated (Table 7). Evidently, the q_e_ value observed in this work was higher than those in the list, suggesting the use of an appropriate adsorption system. However, the regeneration efficiency is somehow relatively low, suggesting the need for future exploratory study for the optimum eluent. Accordingly, the overall finding may suggest the great potential application of PACK in dye removal from aqueous solution.

## 3. Materials and Methods

### 3.1. Materials

Pyrrole monomer C_4_H_5_N (Py, +98%), ammonium persulfate (NH_4_)_2_S_2_O_8_ (APS, 98%), and potassium hydroxide KOH (pellets, 85%) were purchased from Alfa Aesar, Karlsruhe, Germany. Hydrochloric acid HCl (~36%) and ethanol (99.5%) were obtained from Fisher Chemical, Loughborough, UK. Methyl orange sodium salt C_14_H_14_N_3_NaO_3_S (MO, 99.8%) powder was obtained from BDH Chemicals Ltd., Poole, UK and was dried at 100 °C for 1.5 h. All the reagents were used as received without any further treatment unless otherwise stated, and distilled water was used throughout the experimental process.

### 3.2. Adsorbent

The adsorbent, polypyrrole-based activated carbon (PACK), was prepared following a two-step method described elsewhere [4]. The pyrrole (Py) monomer (0.08 mol) was polymerized using APS (0.12 mol) as an oxidant and HCl (1 L, 0.1 M) as a dopant in a cool ice bath. After an extensive washing process using ethanol and water, the obtained dried polypyrrole (PPy) was subjected to chemical activation using KOH (1:4 weight ratio). Therefore, PPy was thoroughly mixed with KOH, homogenized, and carbonized in a horizontal Carbolite MTF 12/38/250 tube furnace (Walf Laboratories, UK) under the following condition: nitrogen atmosphere, heating ramp rate of 3 °C/min; activation time of 2 h, and activation temperature of 650 °C. The carbon obtained was washed several times, severally, using 0.5 M HCl and water until neutrality, followed by drying in an oven at 100 °C overnight.

Scanning electron microscope (SEM) images were obtained using a JSM-6380 LA, JEOL, Tokyo, Japan. The Fourier transform infra-red (FTIR) spectra of PPy, PACK, MO, and MO-loaded PACK (PACK–MO) were recorded using an FTIR spectrometer (Nicolet iS10, Thermo scientific, Madison, WI, USA) equipped with an attenuated total reflection accessory (ATR; diamond crystal), in the range of 4000–500 cm^−1^ with a resolution of 4 cm^−1^ and total scans of 32 per spectrum. Thermogravimetric analysis (TGA) was performed on a Mettler Toledo TGA/DSC 1 Star system (Columbus, OH, USA), in which about 10 mg of the sample was heated from 25 to 1000 °C at 20 °C/min under a nitrogen flow of 10 mL/min. Differential scanning calorimetry (DSC) thermograms were obtained by a Shimadzu DSC 60 system (Shimadzu, Kyoto, Japan). Samples of 6–10 mg were packed in the aluminum DSC pans and heated under nitrogen gas from 30 to 400 °C with a heating rate of 10 °C/min.

### 3.3. Adsorbate

A 1000 ppm stock solution of MO was prepared in distilled water. The working solutions were obtained by successive dilutions to the desired concentrations, and their pH values were measured prior to each application using a Benchtop pH meter (Orion 3 Star, Thermo Scientific, Beverly, MA, USA) and were found to be 6.65 ± 0.05. A standard curve was established employing 0.5, 1, 2, 4, and 8 ppm MO solutions (*R*^2^ = 9959). The sample concentrations were measured using a double beam UV-Vis spectrophotometer (U-2910, Hitachi, Tokyo, Japan) at room temperature (23 ± 2 °C) and λ_max_ of 465 nm.

### 3.4. Batch Adsorption Studies

#### 3.4.1. Batch Adsorption Experiments

The batch adsorption technique was performed on 25 mL MO solutions of 100, 200, 300, and 500 ppm concentrations in a 50-mL Erlenmeyer flask at temperatures of 25, 35, and 45 °C using 0.02 g of the adsorbent (PACK). The adsorption experiments were conducted using a digital shaker (GLF 3017, GmbH, Burgwedel, Germany) with a constant speed of 150 rpm for 24 h to ensure equilibrium. Thereafter, the solutions were filtered for the remaining MO concentration analyses. The data obtained here were used for the adsorption isotherms and thermodynamic analysis. For the adsorption kinetic study, 0.04 g of the PACK adsorbent was added to 500 mL of a 50 ppm MO solution, agitated at 150 rpm and at 23 ± 2 °C. Subsequently, the MO residual concentration was determined spectrophotometrically at the given intervals (0, 5, 15, 30, 45, 75, 135, and 195 min, then after 24 h). All the experiments were carried out in duplicate without pH adjustment, and at least three replicates of measurements were performed and averaged.

#### 3.4.2. Adsorption Data Evaluation

The adsorbent capacity at equilibrium (q_e_, mg/g) and the removal percentage (Re%) were calculated according to Equations (2) and (3):

(2) $$q_{e}=\frac{\left(C_{0}-C_{e}\right)V}{m}$$

(3) Re%=(C0−CeC0)100

where C_0_ and C_e_ (mg/L) are the MO day liquid phase concentrations before and after adsorption, respectively; V (L) and m (g) are the volume of the adsorption solution and the mass of the dry adsorbent used, respectively.

The adsorption isotherm was evaluated using different models, including Langmuir, Freundlich, and Temkin. The linearized forms of these models are shown respectively in Equations (4)–(6):

(4) $$\frac{C_{e}}{q_{e}}=\frac{C_{e}}{q_{m}}+\frac{1}{K_{L}q_{m}}$$

(5) $${\mathrm{lnq}}_{e}={\mathrm{lnK}}_{F}+\frac{1}{n}{\mathrm{lnC}}_{e}$$

(6) $$q_{e}=\frac{\mathrm{RT}}{K_{T}}\mathrm{lnA}+\frac{\mathrm{RT}}{K_{T}}{\mathrm{lnC}}_{e}=\mathrm{BlnA}+{\mathrm{BlnC}}_{e}$$

where q_e_ and C_0_ have the same meanings as in the previous equation; q_m_ (mg/g) is the maximum monolayer capacity; K_L_ (L/mg), K_F_ (mg/g)(L/mg)^1/*n*^, and K_T_ (J/mol) are the Langmuir, Freundlich, and Temkin isotherm constants, respectively; T (K), R (8.314 J/mol·K), and A (L/g) are the absolute temperature, the universal gas constant, and Temkin isotherm equilibrium binding constant, respectively.

However, each model has to report certain characteristics that describe the adsorption behavior, adsorbent surface properties, adsorption capacity, and type of interaction between the adsorbate and the adsorbent. The Langmuir isotherm basically assumes a homogeneous adsorbent surface having identical and fixed number of equivalent sites; monolayer and uniform adsorption processes with no lateral interaction occurs between the adsorbed molecules on adjacent sites [29]. The essential feature of the Langmuir equation is the equilibrium or separation factor (R_L_, dimensionless) that relates to the adsorption shape, and it can be calculated using Equation (7), in which C_0_ (mg/L) is the initial adsorbate concentration. Therefore, R_L_ > 1 indicates an unfavorable monolayer process; RL = 1, linear; 0 < R_L_ < 1, favorable; and R_L_ = 0 irreversible [30,31]:

(7) $$R_{L}=\frac{1}{1+K_{L}C_{0}}$$

The empirical Freundlich isotherm is a multilayer model that describes a non-uniform surface, a heterogeneous and an exponential distribution of active sites, and their energies [32]. The isotherm equation (Equation (5)) defines the adsorption process, showing that the relatively strong binding sites are initially employed, after which the binding strength reduces as the degree of site occupation increases. The Freundlich constant (K_F_) is an approximate indicator of the adsorption capacity. The magnitude of the heterogeneity factor (1/*n*) (also called the energy of distribution) indicates the tendency of the system equilibrium (i.e., sorption/desorption equilibrium) and depends on the temperature and interactions. The value, 1/*n* = 1, implies a linear adsorption isotherm, indicating that the equilibrium is independent of the concentration and that the energy of all sites is indistinguishable; 1/*n* < 0 indicates a normal adsorption (Langmuir); 1/*n* > 1 indicates a cooperative (multi-layer) adsorption; 0.1 < 1/*n* < 1.0 represents a favorable adsorption condition, i.e., becoming more heterogeneous as the value approaches zero [33,34]. The Temkin isotherm model (Equation (6)) contains a factor that explicitly describes the sorbent/sorbate interaction. By ignoring the exceptionally high and low concentrations of the adsorbate, the model assumes that the heat of adsorption of all the molecules in the layer would reduce linearly rather than logarithmically with [35]. The expression, B = RT/K_T_, containing the Temkin (K_T_) equilibrium binding constant, is related to the heat of adsorption, and thus, the maximum binding energy.

Adsorption thermodynamic parameters such as the Gibbs free energy (∆G°), enthalpy (∆H°), and entropy (∆S°) change were calculated using the Van′t Hoff equations [36] as follows:

(8) $${\mathrm{lnK}}_{0}=\frac{\Delta S{}^{\circ}}{R}-\frac{\Delta H{}^{\circ}}{\mathrm{RT}}$$

(9) $$K_{0}=\frac{q_{e}}{C_{e}}$$

(10) $$\Delta G{}^{\circ}=-{\mathrm{RTlnK}}_{0}$$

where K_0_ (L/g) is the apparent equilibrium constant; the other notations have the same meaning as above.

The adsorption mechanism, as well as the rate-controlling steps, may be established via kinetic models. In this study, the kinetics of the adsorption systems were investigated using the PFO, PSO as well as the intra-particle diffusion models, described by Lagergren–Svenska [37], Ho–McKay [38], and Weber–Morris [36,39], respectively. The linear forms of these models can be expressed as Equations (11)–(14):

(11) $$\log\left(q_{e}-q_{t}\right)=\log(q_{e})-\frac{k_{1}t}{2.303}$$

(12) $$\frac{t}{q_{t}}=\frac{1}{k_{2}q_{e}^{2}}+\frac{t}{q_{e}}$$

(13) $$h=k_{2}q_{e}^{2}$$

(14) $$q_{t}=k_{\mathrm{id}}t^{0.5}+C$$

where q_t_ (mg/g) is the amount of MO dye (mg) adsorbed per mass of PACK (g) at time t (min); k_1_ (min^−1^), k_2_ (g/(mg·min)), and k_id_ (mg/L·min^−0.5^) are the rate constants of the PFO, PSO, and the initial rate constant of intra-particle diffusion models, respectively; h (mg/(g·min)) is the initial rate constant given by the second-order rate constant; and C (mg/g) is the *y*-intercept, providing information about the thickness of the boundary layer. The PFO model (Equation (11)) assumes the adsorption rate based on the adsorption capacity. The PSO (Equation (12)) model is based on the assumption that the adsorption is controlled by the chemisorption mechanism, involving valency forces through electron sharing or transfer between the adsorbent and the adsorbate [38,40]. For the intra-particle diffusion model (Equation (14)), the plot of q_t_ versus t^0.5^ is assumed to be linear, passing through the origin; otherwise, some other mechanism may be involved.

### 3.5. Column Adoption Studies

#### 3.5.1. Column Experimental Set-Up

Continuous adsorption experiments were conducted in a glass column of 1 cm internal diameter and 25 cm height at room temperature (23 ± 2 °C) and without solution pH adjustment (pH 6.65 ± 0.05). A portion (0.1 g) of PACK (~0.7 cm layer height) was loaded into the glass column and carefully sandwiched between two supporting layers of glass wool. The MO solution (30 mg/L) was fed from the top (1 mL/min) in a predesigned set-up working under gravity. That is, the MO solution to be treated was stored in an overhead tank, and two valves at the inlet and outlet were used to control the flow rate, which was further permanently monitored (10 ± 0.56 mL per 10 min). Fractions of 10 mL MO were collected and analyzed spectrophotometrically as above. The operation was halted when almost no change in the effluent concentrations was observed. The column regeneration step was carried out using 0.1 M NaOH under similar operation condition as in the sorption experiments. The adsorption experiment was performed in duplicate, and the data were averaged while one trial column-regeneration experiment was accomplished.

#### 3.5.2. Column Data Analysis

The performance of the column is usually evaluated with the concept of the breakthrough curve. Several theoretical models have been used to describe the column breakthrough behavior in continues adsorption, of which Thomas (Th) and Yoon–Nelson (Y–N) are very common. The linearized forms of the two models are given in Equations (15) and (16), respectively.

(15) $$\ln\left(\frac{C_{0}}{C_{e}}-1\right)=\frac{K_{\mathrm{Th}}q_{e}m}{Q}-K_{\mathrm{Th}}C_{0}t$$

(16) $$\ln\frac{C_{e}}{C_{0}-C_{e}}=K_{\mathrm{YN}}t-{\tau K}_{\mathrm{YN}}$$

(17) $$\mathrm{Ce}=\frac{{\mathrm{bt}}^{\alpha }}{c^{\alpha }+t^{\alpha }}$$

where K_Th_ (L/(mg·min)) and K_YN_ (min^−1^) are the constants of the model; Q (mL/min) and τ (min) are the volumetric flow rate and the time required for 50% adsorbate breakthrough, respectively; b, c, α are the three-parameters logistic Hill model. The other parameters have the same meaning as in the adsorption batch method. The Thomas model is one of the most widely used in column performance theory. It is a surface reaction model, assuming that the mass transfer and adsorption-desorption mode flows both the Langmuir model and the second-order reversible reaction kinetics [41]. The model assumes no axial dispersion, and the adsorption is the rate driving force. Yoon–Nelson (Y–N) is a relatively simple theoretical assumption, which does not focus on the adsorbate properties but, rather, it provides a probable statement: that the decreasing rate of the adsorption is directly proportional to adsorbate adsorption and breakthrough. It predicts the column run time before regeneration or when replacement of the column becomes necessary. The major advantage of this model is that few data concerning the type of adsorption, characteristic of the adsorbate, and physical properties of the adsorbent bed are required. The three-parameters logistic (3PL), otherwise called the Hill model, (Equation (17)) is a basic expression used for evaluating the data obtained from certain experiments [42]. Here, the equation was used for the breakthrough curve analysis to examine how steep the curve is as indicated by the value of the Hill coefficient (α) at the time (termed as c) when the effluent concentration (C_e_) is half the influent concentration (b).

## 4. Conclusions

In this work, polypyrrole-based activated carbon (PACK) was prepared, characterized, and its MO removing capability was investigated. The essential properties of the materials under investigation were also analyzed by various analytical techniques. The adsorption of MO onto PACK was found to be a spontaneous monolayer adsorption through a physical process, described better by the Langmuir isotherm and PSO kinetic model with a rate-limiting step mainly controlled by both the film and intra-particle diffusions. The maximum monolayer adsorption capacity, q_m_, of PACK was 520.8 mg/g. Thermodynamic results indicated an exothermic and spontaneous adsorption at all the tested conditions, while ΔG° became less negative with temperature and the concentration increased. In the column operations, the equilibrium adsorption capacity (q_e_), the saturation percentage, breakthrough point, and exhaustion times were found to be 57.21 mg/g, 87%, 90, and 290 min, respectively. The column regeneration indicates a recovery of 73% during 80 min, with 66% desorbed in the first 20 min. Thus, it can be concluded that PACK is a highly effective adsorbent for the removal of dyes from wastewater.

## Figures and Tables

**Table 1:** TGA degradation steps of PACK and PACK–MO.

Material	Step	TGA, T (°C)	DTG, T (°C)	Mass Loss (%)	Notes
PACK	1	25–95	64	5.6	Moisture, volatiles
	2	95–336	296	8.6	Total mass loss = 48.2%, Residue = 51.8%
	3	336–820	767	25.2	
	4	820–1000	-	gradual	
PACK–MO	1	25–95	51	2.1	Moisture, volatiles
	2	95–265	199	19.1	Total mass loss = 61.1%, Residue = 38.1%
	3	265–445	360	15.6	
	4	445–1000	720	gradual	

**Table 2:** Kinetic parameters of the pseudo-first-order (PFO), pseudo-second-order (PSO), and intra-particle diffusion equations for the adsorption of the MO dye on PACK. Adsorbate C_0_ = 50 mg/L, volume = 500 mL, adsorbent = 0.04 g, temperature = 23 ± 2 °C, and agitation speed = 150 rpm.

q_e_. Exp. (mg/g)	Pseudo-First-Order			Pseudo-Second-Order				Intra-Particle Diffusion		
	q_e_ (mg/g)	k_1_ (min^−1^)	*R* ^2^	q_e_ (mg/g)	k_2_ (g/(mg·min))	*R* ^2^	h (mg/(g·min))	k_id_ (mg/(g·min^0.5^))	C (mg/g)	*R* ^2^
143.38	23.67	0.0115	0.9639	143.89	0.0025	0.9996	51.71	1.925	117.98	0.9303

**Table 3:** Parameters obtained from the Langmuir, Freundlich, and Temkin isotherms.

T (K)	Langmuir				Freundlich			Temkin			
	q_m_ (mg/g)	K_L_ (L/mg)	R_L_ (C_0_ (mg/L) = 100, 200, 300, 500)	*R* ^2^	K_F_ (mg/g) (L/mg)^1/n^	1/*n*	*R* ^2^	A (g/mg)	K_T_ (J/mol)	B	*R* ^2^
298	520.8	0.323	0.030, 0.015, 0.010, 0.006	0.9951	221.8	0.176	0.9979	245.58	53.69	46.15	0.9347
308	512.8	0.263	0.037, 0.019, 0.013, 0.008	0.9961	205.6	0.185	0.9915	134.11	53.61	47.77	0.9229
318	497.5	0.244	0.039, 0.020, 0.013, 0.008	0.9962	198.9	0.183	0.9938	124.03	56.72	46.61	0.9322

**Table 4:** Thermodynamic functions ∆G°, ∆H°, and ∆S° of the MO dye adsorbed onto PACK.

C_o_ (mg/L)	T (K)	lnK_0_	ΔG° (kJ/mol)	ΔH° (kJ/mol)	ΔS° (J/mol·K)	*R* ^2^
100	298	8.1962	−20.31	−27.61	−24.87	0.9521
	308	7.6851	−19.68			
	318	7.5027	−19.84			
200	298	4.7303	−11.72	−29.27	−59.48	0.8911
	308	4.1101	−10.52			
	318	3.9995	−10.57			
300	298	3.0944	−7.667	−7.974	−0.85	0.8479
	308	3.0573	−7.829			
	318	2.8881	−7.636			
500	298	1.6840	−4.172	−9.210	−16.844	0.9849
	308	1.5821	−4.051			
	318	1.4489	−3.831			

**Table 5:** Column parameters from the Thomas, Yoon–Nelson, and Hill models. C_0_ = 30 mg/L, flow rate (Q) = 1.0 mL/min, and temperature = 23 ± 2 °C.

q_e_. Exp. (mg/g)	Thomas			Yoon–Nelson			3-PL Hill			
	K_Th_ (L/(mg·min))	q_e_ (mg/g)	*R* ^2^	K_YN_ (L/(mg·min))	τ (min)	*R* ^2^	c	b	α	*R* ^2^
57.21	0.858	69.71	0.9676	0.0286	209.12	0.9676	219.8	1.17	3.67	0.9968

**Table 6:** Comparison of the adsorption capacities of various adsorbents for MO removal.

Adsorbent	q_m_ (mg/L)	Adsorption Conditions				Reference	Source Type	Reusability
		Co (mg/L)	pH	T (°C)	Adsorbent Dosage (g/L)			
Polyaniline microspheres	154.6	-	-	RT	2.0	[15]	Synthetic	-
Banana peels	21.0	100	5.7	30	1.0	[24]	Agriculture	-
Orange peels	20.5	100	5.7	30	1.0	[24]	Agriculture	-
Hyper crosslinked polymer	70.9	100	-	20	2.5	[2]	Synthetic	-
NH_3_^+^-MCM-41	366.6	98.2	5.6	24.5	0.5	[25]	Synthetic	-
PANI/CPL	333.3	50	4.0	-	2.0	[26]	Synthetic	-
Organo-modified silkworm exuviae (MSE)	87.03	300	-	30	2.0	[26]	Biosource waste	17.25%
surfactants modified montmorillonite	128.21	120	-	30	2.0	[6]	Modified-Natural source	11.5%
PACK	520.8	100–500	6.5	25	0.8	This work	Synthetic	-

**Table 7:** Comparison of column equilibrium capacity of various adsorbents for MO removal.

Adsorbent	Column Conditions				q_e_ (mg/g)	*R* ^2^	Reference	Regeneration
	C_0_ (mg/L)	pH	Flow Rate (mL/min)	Bed (cm)				
Iron oxide-coated zeolite (IOCZ)	30	2.5	11	6	0.334	0.772	[5]	-
Waste tyre activated carbon (WTAC)	40	-	3	1.5	6.89	0.983	[27]	89%
De-oiled Soya	33	6.5	0.5	1 g	3.5	-	[28]	93%
PACK	30	6.5	1.1	0.7	69.1	0.9676	This work	73%

## Supplementary Materials

The following are available. Figure S1: Scanning electron microscope (SEM) of PPy (**left**) and PACK (**right**); Figure S2: EDX results of PPy elemental composition; Figure S3: EDX results of PPy elemental composition. Table S1: Elemental composition of PPy and PACK.
