# Supplementary material for: Adsorption of Azo Dye Methyl Orange from Aqueous Solutions Using Alkali-Activated Polypyrrole-Based Graphene Oxide
Source: Molecules. 2019 Oct 13;24(20):3685. doi: 10.3390/molecules24203685 (PMC6833077; doi:10.3390/molecules24203685)
Supplement: Supplementary file 1 [file molecules-24-03685-s001.pdf]

molecules

MDPI

Supplementary Materials

Adsorption of Azo Dye Methyl Orange from Aqueous Solutions Using Alkali-Activated Polypyrrole-Based Graphene Oxide

Abdulaziz Ali Alghamdi, Abdel-Basit Al-Odayni *, Waseem Sharaf Saeed *, Mohammed S. Almutairi, Fahad A. Alharthi, Taieb Aouak and Abdullah Al-Kahtani

Chemistry Department, College of Science, King Saud University, P.O. Box 2455, Riyadh 11451, Saudi Arabia; aalghamdia@ksu.edu.sa (A.A.A.); 436105200@student.ksu.edu.sa (M.S.A.); fharthi@ksu.edu.sa (F.A.A.); taouak@ksu.edu.sa (T.A.); akahtani@ksu.edu.sa (A.A.-K.)

* Correspondence: aalodayni@ksu.edu.sa (A.-B.A.-O.); wsaeed@ksu.edu.sa (W.S.S.); Tel.: +966-11-469-9889

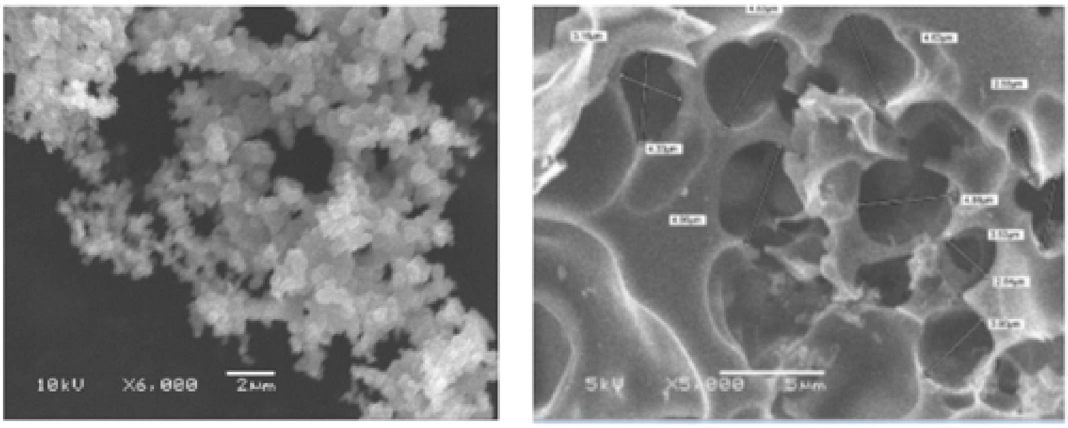

Figure S1. Scanning electron microscope (SEM) of PPy (**left**) and PACK (**right**).

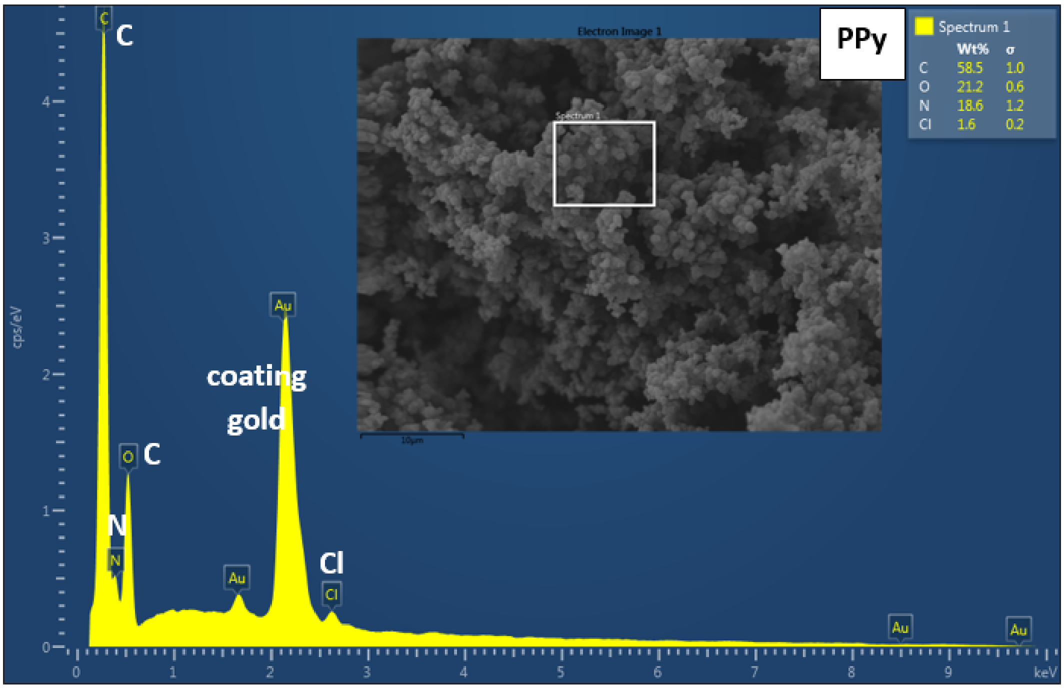

Figure S2. EDX results of PPy elemental composition.

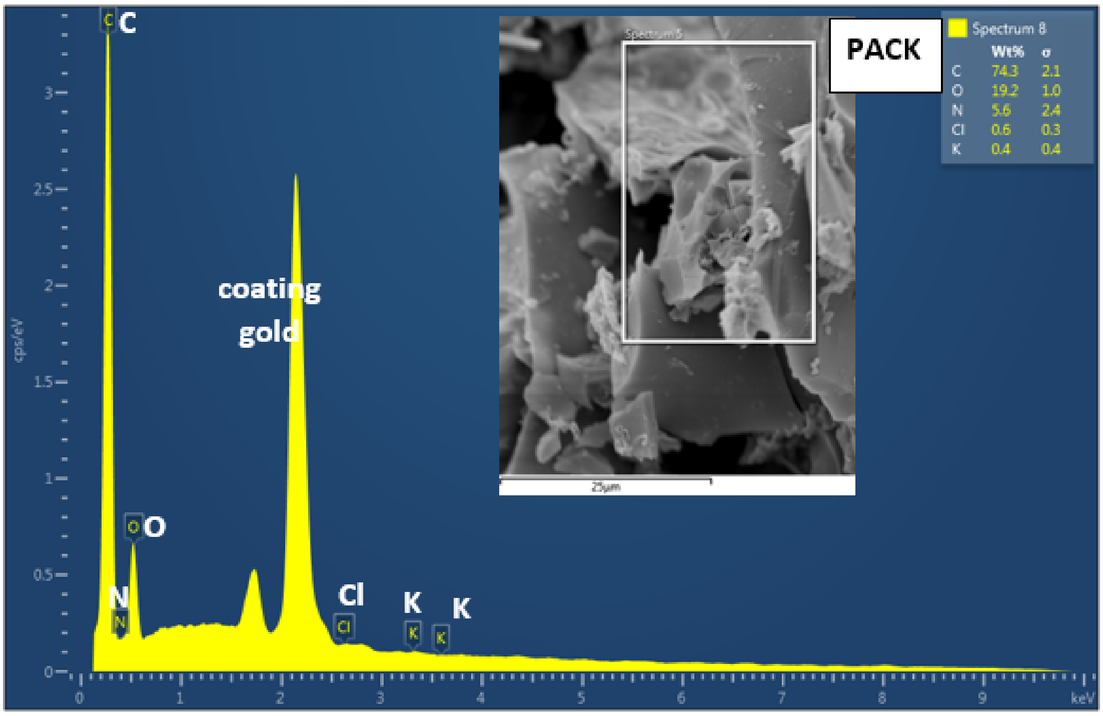

Figure S1. EDX results of PPy elemental composition.

Table S1. Elemental composition of PPy and PACK.

Sample	Yield %	Elemental Composition wt%						Reference
		N	C	O	H	Cl	K	
PPy	98.9	11.62	77.52	8.81	1.27	-	-	[15]
		16.67	57.22	22.01*	4.10	-	-	This work (CHN)
		20.05	58.43	20.00	-	1.58	0.00	This work (EDX)
PACK	5.1	1.45	79.99	16.95	1.53	-	-	[15]
		5.69	64.16	28.31*	0.94	-	-	This work (CHN)
		4.70	73.17	20.37	-	0.50	0.40	This work (EDX)

* calculated values assuming total compositions of 100%.
